# A spontaneous transomental hernia through the greater omentum

**DOI:** 10.11604/pamj.2015.20.384.6432

**Published:** 2015-04-17

**Authors:** Anisse Tidjane, Benali Tabeti, Nabil Boudjenan Serradj, Abdessamed Djellouli, Noureddine Benmaarouf

**Affiliations:** 1 Department of Hepatobiliary Surgery and Liver Transplantation, EHU-1st November 1954, Oran, Algeria

**Keywords:** Internal hernia, transomental hernia, acute intestinal obstruction

## Abstract

Transomental hernia is the rarest form of internal hernias. Clinical expression of this pathology is ambiguous and diagnosis is often made at complication phase, after irreversible strangulation of the herniated loop. Radiological diagnosis is still difficult and intraoperative exploration usually allows discovering this pathology when patient is operated for acute intestinal obstruction. Treatment is surgical and aims to treat intestinal obstruction and prevent recurrence. We describe the case of a 65 years old male operated for a preoperatively suspected internal hernia; surgical exploration found a transomental hernia trough the greater omentum.

## Introduction

Adult's Internal hernias (IH) are rare [1, 2], they match to the protrusion of the intestine through a congenital or acquired intraperitoneal aperture [1–3], and must be differentiated from postoperative internal hernias. IH represent less than 1% of all acute intestinal obstruction (AIO) [1, 3, 4]. Even the advances in imaging technics, diagnosis of this disease remains difficult and it is still often made during the operative time [3, 5, 6]. Transomental internal hernia (TOH) is the rarest type, and represents 1 to 4% of the IH [1, 3, 5, 7]. It interests mostly the greater omentum and exceptionally the gastrocolic ligament or the lesser omentum [1]. We describe the case of a 65 years old male operated for a preoperatively suspected IH. Surgical exploration found a TOH trough the greater omentum.

## Patient and observation

**Figure 1:**
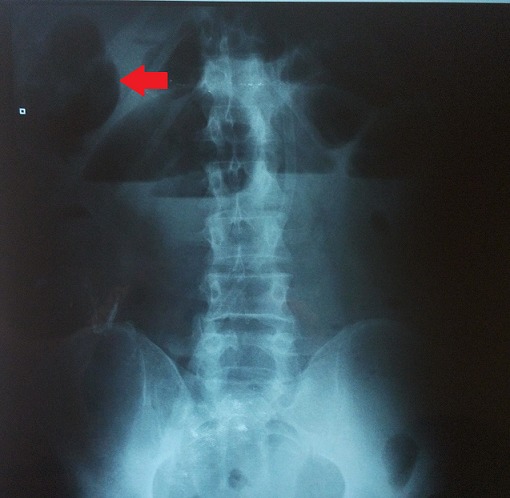
Standard radiography of the abdomen, note the small air fluid level projected upper the right quadrant

**Figure 2:**
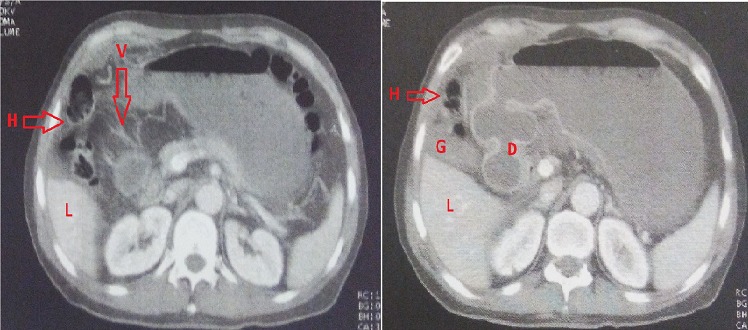
Abdominal CT scan, G= gallbladder, R= right Liver, D= duodenum, H = herniated loop, V= mesenteric vessels

**Figure 3:**
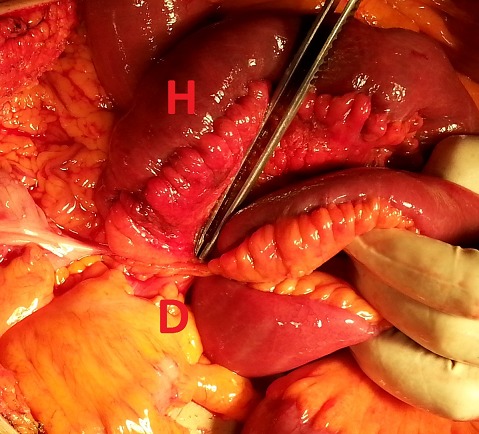
Intra-operative view, D= departure, H= herniated ileal loop

**Figure 4:**
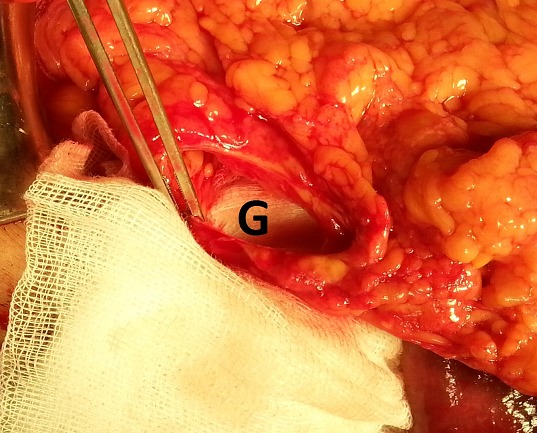
Intra-operative view, G= the greater omentum defect after bowel reduction

We report the case of a 65 years old Algerian male, without any surgical or medical history, except the antecedent of recurrent paroxystic abdominal pain, and constipation ceding quickly and spontaneously without any treatment, explored by colonoscopy and abdominal CT but no organic pathology was found. Patient was admitted this time for abdominal pain, vomiting and permanent constipation evolving since less than 24 hours. At admission time the patient was in good conditions, conscious and cooperating, blood pressure was 100/60 mmhg, physical examination showed an average central abdominal distension with no more abnormality. Biology tests founds, leukocytosis at 12,000 elements/mm^3^, blood urea at 0.60 g/l and sodium at 131 meq/l. A standard radiography of the abdomen showed many small and central air-fluid levels, with an abnormal projection of an air-fluid level near to the liver area (Figure 1). An abdominal CT scan confirmed the presence of a small bowel loop with its mesenteric vessels projected between the right colon and the liver (Figure 2). The diagnosis of acute intestinal obstruction caused by a spontaneous internal hernia was suspected without precision of its type. Patient was operated through a midline laparotomy. Operative exploration showed a TOH trough a defect in the right side of the greater omentum measuring 4 cm of diameter strangling a small bowel loop that measured 40 cm, and looked ischemic but returned back to normal coloration after reduction (Figure 3, Figure 4). To prevent recurrences we proceeded to the section of the greater omentum from its free edge to the abnormal defect. No particular event occurred in postoperative period, and patient was discharged on day 3.

## Discussion

IH are rare, the most common form of IH is the para-duodenal hernia, while the TOH is the rarest form and represents 1-4% of all IH, and the TOH has a collet but no hernia sac [1–3]. Diagnosis of TOH is usually made after the age of 50 years [3, 6]. Hernia defect is congenital or acquired, post-traumatic, secondary to intraperitoneal inflammatory disease or due to the appearance of area of weaknesses related to age in the greater omentum. In few cases the patient is young without any surgical or traumatic history and free from any inflammatory disease of the peritoneum, in this exceptional case the TOH is spontaneous [3–7]. Diameter of the defect on the greater omentum varies from 2 to 10 cm [3, 6], through which protrudes a part of the small bowel or occasionally another free segment of intestine as cecum or sigmoid colon [3, 6]. Clinical manifestations are unspecific causing diagnosis delay [2]. Patients describe recurrent and ambiguous symptoms, combining paroxysmal abdominal pain, nausea, bloating and constipation that disappear spontaneously [3, 4, 7]. In more acute and irreversible forms the patient consults for AIO which is the manifestation of complicated form [1, 4, 6]. Complicated internal hernias represent less than 1% of all the AIO [1, 3], radiography of the abdomen made in this emergency circumstances shows nonspecific central and small air-fluid level caused by the small bowel obstruction. In rare cases an abnormal position of a small air-fluid which is the consequence of an abnormal protrusion of the small bowel is viewed [7]. Modern imaging represented by abdominal multidetector computed tomography (CT) allows in 16% of cases the diagnosis of TOH [5], by viewing the passage of mesenteric vessels through the greater omentum with a stretched loop in an abnormal position in the right paracolic gutter when the defect interests the greater omentum [6], or an abnormal bowel loops retropositioned between the stomach and the pancreas when the defect interests the gastro-colic ligament [5]. Difficulty of the diagnosis of TOH is explained by the inexistence of an hernia sac making it more difficult than other forms of IH. Up to the present day, diagnosis of TOH is still intraoperative [3, 5, 6]. Treatment of TOH is surgical, approached by laparoscopy or laparotomy [7], the herniated bowel must be reduced [1, 6], and the omental defect has to be closed to prevent recurrences [1, 7], section of the greater omentum from its free edge to the abnormal defect is an alternative [6]. Total omentectomy is performed if exploration shows pathological greater omentum [1, 7]. In case of intestinal necrosis, bowel resection is necessary with respect for traditional rules of digestive continuity restoration [1].

## Conclusion

Internal hernia should be suspected in any patient with antecedent of paroxysmal abdominal pain and hospitalized for acute intestinal obstruction. Despite the advances in radiology, diagnosis is still made during operative time, this is due to the diversity of these hernias, to their misconception and especially to the urgent character of the acute intestinal obstruction forcing surgeons to operate as soon as possible and not having time to ask for modern radiology and appreciate its contribution.
